# Effect of carbamazepine and levetiracetam on coagulation parameters: Prothrombin time, activated partial thromboplastin time, D-dimer, and fibrinogen levels

**DOI:** 10.5937/jomb0-52052

**Published:** 2025-07-04

**Authors:** Jiaohui Li, Chengyun Zhong, Guanji Bian

**Affiliations:** 1 Cancer Hospital of the University of Chinese Academy of Sciences (Zhejiang Cancer Hospital), Taizhou Branch, Department of Neurology, Taizhou, Zhejiang Province, China; 2 Taizhou Cancer Hospital, Department of Neurology, Taizhou, Zhejiang Province, China; 3 Traditional China Medical Hospital of Zhuji, Department of Neurology, Zhuji, Zhejiang Province, China

**Keywords:** partial-onset epilepsy, carbamazepine, levetiracetam, coagulation factors, frequency of epileptic seizures, parcijalni epileptišnih napadi, karbamazepin, levetiracetam, koagulacioni faktori, učestalost epileptišnih napada

## Abstract

**Background:**

Epilepsy is a prevalent neurological disorder, and evaluating its treatment strategies is highly significant. This study aimed to compare the effects of monotherapy with carbamazepine and levetiracetam on the results of coagulation tests, including prothrombin time, activated partial thromboplastin time, fibrinogen, and D-dimer levels, as well as on seizure control in patients with partial-onset epilepsy.

**Methods:**

A total of 89 patients diagnosed with POE and treated at our hospital between January 2023 and January 2024 were enrolled. The patients were divided into the carbamazepine group and the levetiracetam group. Blood coagulation parameters, including prothrombin time, activated partial thromboplastin time, fibrinogen, and D-dimer levels, were measured at baseline (before treatment) and at 1, 3, and 6 months after medication initiation. Additionally, the frequency and severity of epileptic seizures were recorded for each group.

**Results:**

In the carbamazepine group, prothrombin time, activated partial thromboplastin time, and D-dimer levels were significantly reduced at 1-, 3-, and 6-months post-treatment compared to pre-treatment levels. Conversely, these changes were less pronounced in the levetiracetam group. Fibrinogen levels decreased in both groups after treatment. The frequency of epileptic seizures was markedly reduced in all patients after treatment. There was no significant difference in seizure control rates between the carbamazepine and levetiracetam groups.

**Conclusions:**

Carbamazepine may pose a higher risk of coagulation abnormalities but demonstrated strong efficacy in controlling epileptic seizures. Levetiracetam had a milder impact on coagulation parameters while offering comparable effectiveness in seizure management.

## Abstract (sr)

**Uvod:**

Epilepsija je čest neurološki poremećaj, a procena strategija njenog lečenja ima veliki značaj. Ova studija imala je za cilj da uporedi efekte monoterapije karbamazepinom i levetiracetamom na rezultate koagulacionih testova, uključujući protrombinsko vreme, aktivirano parcijalno tromboplastinsko vreme, nivoe fibrinogena i D-dimera, kao i na kontrolu napada kod pacijenata sa parcijalnim epileptiškim napadima.

**Metode:**

U istraživanje je uključeno ukupno 89 pacijenata sa dijagnozom parcijalnih epileptišnih napada, lečenih u našoj bolnici u periodu od januara 2023. do januara 2024. godine. Pacijenti su podeljeni u grupu koja je primala karbamazepin i grupu koja je primala levetiracetam. Parametri koagulacije, uključujući protrombinsko vreme, aktivirano parcijalno tromboplastinsko vreme, nivoe fibrinogena i D-dimera, mereni su na početku (pre terapije) i nakon 1, 3 i 6 meseci od započinjanja terapije. Takođe su zabeležene učestalost i težina epileptišnih napada u svakoj grupi.

**Rezultati:**

U grupi koja je primala karbamazepin, protrombinsko vreme, aktivirano parcijalno tromboplastinsko vreme i nivo D-dimera značajno su smanjeni nakon 1, 3 i 6 meseci terapije u poređenju sa vrednostima pre terapije. Nasuprot tome, ovi parametri su pokazali manje izražene promene u grupi koja je primala levetiracetam. Nivoi fibrinogena su opali u obe grupe nakon terapije. Učestalost epileptišnih napada se značajno smanjila kod svih pacijenata posle terapije, bez značajne razlike u efikasnosti kontrole napada između grupa koje su primale karbamazepin i levetiracetam.

**Zaključak:**

Karbamazepin može da predstavlja veći rizik za poremećaje koagulacije, ali je pokazao visoku efikasnost u kontroli epileptičnih napada. Levetiracetam je imao blaži uticaj na koagulacione parametre, dok je pružao sličnu efikasnost u upravljanju epilepsijom.

## Introduction

Epilepsy is a prevalent neurological disorder characterized by recurrent, abnormal electrical activity in the brain, leading to seizures. Approximately 1-2% of the global population is affected by epilepsy, with a substantial number of cases manifesting in childhood [1] [2] [3] [4] [5] [6]. Epilepsy is classified into two main categories: generalized and partial-onset epilepsy (POE). POE originates from a localized region of the brain, resulting in focal seizures that may or may not evolve into generalized seizures [4] [5] [6] [7] [8] [9]. Various underlying conditions, such as brain injuries, congenital malformations, cerebrovascular diseases, infections, and neurodegenerative disorders, can trigger the onset of epilepsy [10] [11] [12] [13] [14] [15].

The management of epilepsy predominantly involves pharmacological treatments, with surgical options reserved for patients unresponsive to medication [16]. Among the most commonly prescribed antiepileptic drugs (AEDs) are carbamazepine (CBZ) and levetiracetam (LEV), both used for managing partial-onset seizures. However, while these drugs are effective in controlling seizures, their potential impact on coagulation parameters warrants further investigation.

Carbamazepine, a first-line treatment for POE, exerts its anticonvulsant effect by inhibiting voltage-gated sodium channels, thus preventing abnormal neuronal firing [17]. Additionally, CBZ has been shown to interact with other ion channels, including voltage-gated calcium channels, contributing to its anticonvulsant properties [18]. On the other hand, levetiracetam operates by enhancing the effects of the neurotransmitter γ-aminobutyric acid (GABA), which plays a crucial role in inhibiting neuronal excitability and thereby reducing seizure occurrence [10] [19]. While these medications effectively control seizures, both have been linked to potential changes in coagulation function.

Coagulation factors are crucial for maintaining hemostasis, including prothrombin time (PT), activated partial thromboplastin time (APTT), fibrinogen, and D-dimer levels. Any alterations in these parameters could predispose patients to bleeding or thrombotic events, complicating the management of epilepsy. Several studies have examined the effects of AEDs on coagulation, revealing that drugs like CBZ and LEV may influence coagulation pathways. For example, CBZ has been associated with thrombocytopenia and alterations in platelet function [20], while LEV has been linked to a reduction in fibrinogen binding and decreased platelet aggregation [21] [22] [23]. These findings underscore the need for closer monitoring of coagulation parameters in patients receiving these drugs [24].

In clinical practice, patients with epilepsy often undergo routine coagulation tests, especially when they are prescribed AEDs. Understanding how CBZ and LEV affect these tests is essential, as they can influence clinical decisions, including managing comorbid conditions. Despite their widespread use, limited research has been conducted on the direct impact of these two drugs on coagulation parameters, particularly in patients with POE. Previous studies have shown that medications such as adrenocorticotropic hormone (ACTH) can alter coagulation profiles in other pediatric conditions [25]. Yet, the effects of AEDs like CBZ and LEV remain less explored.

The primary aim of this study is to investigate the effects of monotherapy with CBZ and LEV on coagulation parameters, specifically PT, APTT, fibrinogen, and D-dimer, in patients with partial-onset epilepsy. Additionally, the study will examine the relationship between these coagulation changes and seizure control, providing valuable insights for clinical management. Given that both drugs are commonly prescribed for POE, understanding their impact on coagulation tests is crucial for optimizing patient care and minimizing potential risks [26].

This study also aims to provide further evidence on the safety and efficacy of CBZ and LEV in treating epilepsy while considering their effects on blood coagulation. By comparing these two AEDs regarding their influence on coagulation tests, we hope to offer clinicians helpful guidance for selecting the most appropriate treatment for patients with POE. Ultimately, the goal is to enhance patient outcomes by providing a comprehensive understanding of the potential risks and benefits of these drugs in coagulation and seizure control [27] [28].

While carbamazepine and levetiracetam are both effective in controlling epileptic seizures, their impact on coagulation parameters needs to be carefully considered. Alterations in PT, APTT, fibrinogen, and D-dimer levels could potentially complicate the management of epilepsy in certain patients. Therefore, this study seeks to fill a critical gap in the literature by evaluating the effects of these AEDs on coagulation, ultimately supporting informed decision-making in the treatment of partial-onset epilepsy.

## Materials and methods

### Research objects

In this work, 88 patients with POE diagnosed and treated at our institution from January 2023 to January 2024 were enrolled and assigned into two groups using a random number table method: the CBZ group and the LEV group, with 44 patients in each.

Patients enrolled had to satisfy the following conditions: patients met the latest classification criteria of the *International League Against Epilepsy* (ILAE); patients aged 16 years and above; patients who had been taking antiepileptic drugs for the past two weeks; patients with a frequency of epileptic seizures greater than 1 time per month; and patients who provided informed consent.

Patients with any of the following conditions had to be excluded: patients with a history of substance abuse; patients with refractory epilepsy; patients with organ dysfunction; patients with neurological disorders preventing them from expressing their thoughts; pregnant patients; and patients resistant to the study medication used in this research.

The study received approval from our institution’s ethics committee, and patients consented to sign informed consent forms.

### Treatment programs and dosage arrangement

Patients in the CBZ group were administered CBZ tablets (SFDA Approval Number: H23021196, Manufacturer: Harbin Pharmaceutical Group Pharma ceutical Factory, Specification: 0.1 g). The initial dosage was set at 0.1 g per dose once daily. After 7 days, the dosage was increased to 0.2 g per dose three times daily. Adjusting the dosage every 7 days based on individual patient conditions and maintaining it below 0.4 g per dose was recommended. The total treatment duration was 6 months.

Patients in the LEV group were administered LEV tablets (State Food and Drug Administration (SFDA) Approval Number: H20223193, Manu fac turer: Yangtze River Pharmaceutical Group Nanjing Hanling Pharmaceutical Co., Ltd., Specification: 0.5 g). The initial dosage was set at 10 mg per kilogram per day, taken twice daily. After 14 days, the dosage was increased to 15 mg per kilogram daily, also taken twice daily. It was recommended to increase the dosage by 5 mg per kilogram daily every 14 days, and maintain it at or below 35 mg per kilogram daily. The total treatment duration was 6 months.

### Observation parameters

After patient enrollment, fasting venous blood samples of 6 mL were collected from each patient at baseline and 1, 3, and 6 months after the commencement of medication. Blood was drawn into 3.2% sodium citrate tubes (e.g., BD Vacutainer, 3.2% sodium citrate, 5 mL volume) to prevent coagulation. The samples were then aliquoted into 1 mL Eppendorf tubes and stored at -80°C for later analysis.

For coagulation tests, the following parameters were assessed: Prothrombin Time (PT), Activated Partial Thromboplastin Time (APTT), Fibrinogen, and D-Dimer. The specific analytical methods and reagents used are outlined below:

1. **Prothrombin Time (PT)**

**Reagent**: Innovin® Thromboplastin Reagent

**Manufacturer**: Siemens Healthineers

**Measurement Unit**: Seconds (s) and International Normalized Ratio (INR)

2. **Activated Partial Thromboplastin Time (APTT)**

**Reagent**: Actin® FS

**Manufacturer**: Siemens Healthineers

**Measurement Unit**: Seconds (s)

3. **Fibrinogen**

**Reagent**: Clauss Method Reagent

**Manufacturer**: Instrumentation Laboratory (IL)

**Measurement Unit**: mg/dL or g/L

4. **D-Dimer**

**Reagent**: D-Dimer ELISA Kit

**Manufacturer**: BioVendor

**Measurement Unit**: μg/mL

In addition to coagulation testing, patients were evaluated for depressive symptoms each month before and after medication using the Hamilton Anxiety Scale (HAMA) and the Hamilton Depression Rating Scale (HAMD) [29]. Cognitive function was assessed using the Chinese Revised Wechsler Adult Intelligence Scale (WAIS-RC), which measures intelligence across various domains such as knowledge, computational ability, comprehension, numerical breadth, and picture arrangement [24].

The accuracy of the electroencephalogram (EEG) results was compared with the frequency of epileptiform discharges. Discharge frequency was categorized based on the following diagnostic criteria: no change (no observable difference in epileptiform discharges compared to before treatment), practical (discharges reduced by less than 50%), remarkably effective (discharges reduced by more than 50%), and restored to normal (discharges disappeared).

### Detection methods of coagulation parameters in the blood

Patients’ PT, APTT, and D-D were measured using the CX-9000 fully automated coagulation analyzer (Mindray Bio-Medical), while FIB levels were determined using the von-Clauss method.

### Assessment method for frequency and severity of epileptic seizures

The treatment effects were compared 1, 3, and 6 months after medication administration. Diagnostic criteria were as follows: Significant control was defined as a reduction of 70% or more in the frequency of epileptic seizures, accompanied by significant alleviation of symptoms. Partial control was characterized by a reduction of 30% or more in the frequency of epileptic seizures, with mild alleviation of symptoms. Ineffectiveness was denoted by no change in symptoms and a decrease in epileptic seizure frequency of less than 30%.

### Statistical analysis

Clinical data of patients were organized and processed using SPSS 26.0. The number of individuals or cases was expressed as percentages (%), and the chi-squared (SIMBOL) test was employed for comparisons. Results were presented as mean ± standard deviation for data that followed a normal distribution, with the t-test used for comparisons. The median and interquartile range (IQR) were used for the presentation of data that did not follow a normal distribution. Statistical significance was considered when P<0.05.

## Results

### Demographic and baseline characteristics

The study included 88 patients with partialonset epilepsy (POE), randomly assigned to the carbamazepine (CBZ) group (n=44) and the levetiracetam (LEV) group (n=44). The median age of patients in the CBZ group was 37 years (range: 28-45 years), and in the LEV group, it was 38 years (range: 30–46 years). The median duration of illness (DOI) was 4 years (range: 3–6 years) in both groups. No statistically significant differences were observed in sex distribution, types of epilepsy (e.g., genetic, post-stroke, infectious), or other baseline clinical characteristics between the two groups (p>0.05). These similarities ensured a well-matched population for comparative analysis.

### Changes in coagulation parameters

Table 1 summarizes the changes in coagulation parameters, including prothrombin time (PT), activated partial thromboplastin time (APTT), fibrinogen (FIB), and D-dimer levels, before and after treatment.

**Table 1:** Baseline characteristics. Note: * indicated an obvious difference with P<0.05 between the conditions in various groups.

Group	Normal	Remarkably<br>effective	Effective	Ineffective	TER	Increased frequency of<br>epileptiform discharges
CBZ group	9	17	10	8	36(81.81%)	6(13.63%)
LEV group	13	16	11	4	40(90.90%)*	2(4.54%)*
χ^2^					7.395	2.843
* P *					<0.05	<0.05

- **CBZ Group**: Significant reductions in PT, APTT, and D-dimer levels were observed at 1, 3, and 6 months after treatment compared to baseline (p<0.05 for all). These results suggest that CBZ monotherapy has a marked impact on coagulation function, potentially increasing the risk of coagulation-related abnormalities over time.
- **LEV Group**: In contrast, there were no statistically significant changes in PT, APTT, or D-dimer levels at any time point compared to baseline (p>0.05), indicating a minimal effect on coagulation function.
- FIB Levels: A significant reduction in FIB levels was noted in both groups at 1-, 3-, and 6 months post-treatment compared to baseline (p<0.05). However, the magnitude of reduction was comparable between the CBZ and LEV groups, with no significant differences observed at any time point (p>0.05).

These findings highlight the differential impact of CBZ and LEV on coagulation parameters, with CBZ showing more pronounced effects.

### Psychological and cognitive assessments

- **HAMA Scores**: Patients in the LEV group experienced a significant increase in Hamilton Anxiety Scale (HAMA) scores during the first two months of treatment (p<0.05), likely reflecting an initial exacerbation of anxiety symptoms. However, these scores gradually returned to baseline levels by the third month. The CBZ group showed no significant changes in HAMA scores throughout the treatment period (p>0.05). Between-group comparisons indicated significantly higher HAMA scores in the LEV group during the first two months of treatment (p<0.05).
- **HAMD Scores**: Both groups exhibited slight fluctuations in Hamilton Depression Rating Scale (HAMD) scores, with no significant differences observed compared to baseline or between the two groups (p>0.05). These results suggest that neither CBZ nor LEV had a substantial impact on depressive symptoms throughout treatment.
- **Cognitive Function**: Cognitive performance, as assessed by the Wechsler Adult Intelligence Scale-Revised (WAIS-RC), showed significant improvement in both groups at 6 months post-treatment compared to baseline (p<0.05). The observed increases in WAIS-RC scores suggest that CBZ and LEV contributed to enhanced cognitive function. However, the two groups had no significant differences in cognitive outcomes (p>0.05).

### Seizure control and EEG findings

- **Seizure Frequency**: Significant seizure control, defined as a reduction of 70% in seizure frequency, was achieved in 52.27% of patients in the CBZ group and 59.09% of patients in the LEV group. Partial control (reduction of 30-69%) was achieved in 38.63% of CBZ-treated patients and 34.09% of LEV-treated patients. Only a small percentage of patients in both groups showed no improvement in seizure frequency (CBZ: 9.09%, LEV: 6.82%). The overall seizure control rates (CBZ: 90.90%, LEV: 93.18%) were comparable between the groups (p>0.05).
- **EEG Results**: EEG analysis revealed a higher total effective rate (TER) of improvement in the LEV group (90.90%) compared to the CBZ group (81.81%, p<0.05). Additionally, the frequency of epileptiform discharges increased in 13.63% of patients in the CBZ group, which was significantly higher than the 4.54% observed in the LEV group (p<0.05). These findings indicate that while both drugs were effective in controlling seizures, LEV may have a more favourable impact on EEG outcomes.

The results demonstrate that CBZ and LEV are both effective in reducing seizure frequency and improving cognitive function in patients with POE. However, CBZ treatment was associated with significant alterations in coagulation parameters, particularly reductions in PT, APTT, and D-dimer levels, which may have clinical implications for long-term use. In contrast, LEV had minimal effects on coagulation but showed better tolerability in terms of anxiety-related side effects and EEG improvements. These findings underscore the importance of individualized treatment selection based on patient-specific risks and therapeutic goals.

## Discussion

This study investigated the effects of carbama zepine (CBZ) and levetiracetam (LEV) monotherapy on coagulation parameters in patients with partialonset epilepsy (POE). Our findings highlight significant differences in the impact of these antiepileptic drugs (AEDs) on coagulation tests, contributing to understanding their broader clinical implications.

The results showed that CBZ treatment was associated with a significant reduction in prothrombin time (PT), activated partial thromboplastin time (APTT), and D-dimer levels over 6 months, suggesting that CBZ may alter coagulation pathways and increase the risk of coagulation-related abnormalities. These findings are consistent with previous studies that have reported CBZ’s influence on coagulation factors, such as its association with thrombocytopenia and alterations in platelet function [2]. Additionally, CBZ’s effects on PT and APTT levels have been linked to its induction of hepatic enzymes, which accelerate the metabolism of vitamin K-dependent clotting factors [4].

In contrast, LEV showed minimal effects on PT, APTT, and D-dimer levels, indicating a safer coagulation function profile. However, FIB levels decreased significantly in both the CBZ and LEV groups, which may reflect a common underlying mechanism in antiepileptic drug action that warrants further investigation. These results align with prior research suggesting that LEV has a limited impact on coagulation, with its primary adverse hematologic effects being mild and infrequent, such as leukopenia [18].

**Figure 1:**
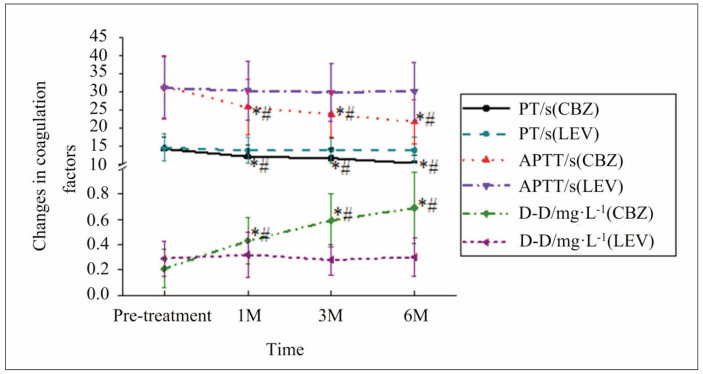
Changes in coagulation factors of patients. Note: * suggested a great difference with P<0.05 between the pre-treatment and post-treatment levels, and # indicated an obvious difference with P<0.05 between the levels in various groups.

**Figure 2:**
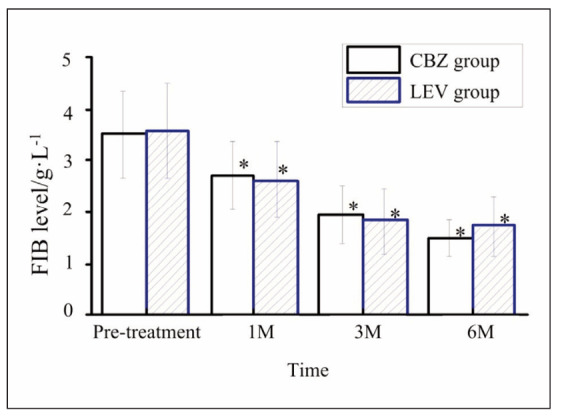
Changes in FIB content of patients at various time points. Note: * suggested a great difference with P<0.05 between the pre-treatment and post-treatment content.

**Figure 3:**
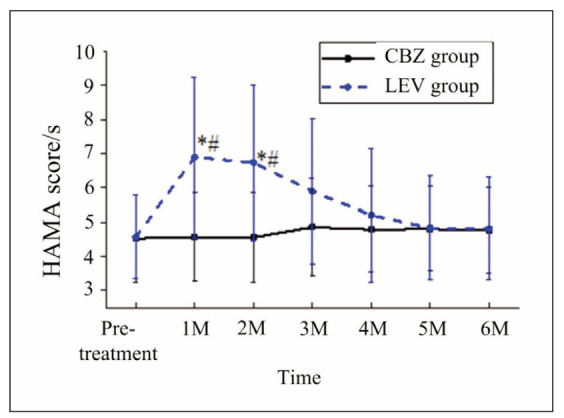
Changes in HAMA scores of patients before and after administration. Note: * suggested a great difference with P<0.05 between the pre-treatment and post-treatment levels, and # indicated an obvious difference with P<0.05 between the levels in various groups.

The observed differences between CBZ and LEV in their impact on coagulation may be explained by their distinct mechanisms of action. CBZ is a potent inducer of cytochrome P450 enzymes, which can disrupt normal coagulation processes, whereas LEV’s pharmacodynamic effects are more localized to synaptic vesicle glycoprotein 2A (SV2A), resulting in minimal systemic metabolic interactions [27]. Figure 1 Figure 2 Figure 3

The significant alterations in coagulation parameters observed with CBZ treatment underscore the need for regular monitoring of coagulation function in patients receiving this drug, particularly those at risk for bleeding or thrombotic events. In clinical practice, this could involve periodic PT and APTT testing, along with careful consideration of alternative treatments such as LEV for patients with pre-existing coagulopathy or a history of thromboembolic disorders.

**Figure 4:**
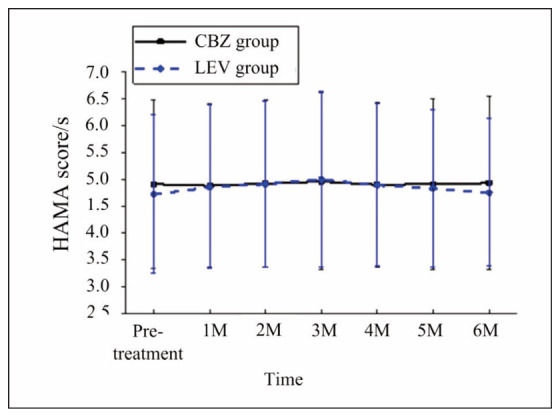
Changes in HAMD scores of patients before and after administration.

**Figure 5:**
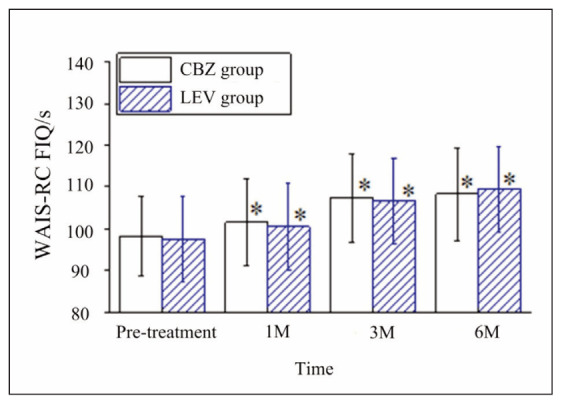
Changes in WAIS-RC scores of patients before and after administration. Note: * suggested a great difference with P<0.05 between the pre-treatment and post-treatment score.

**Figure 6:**
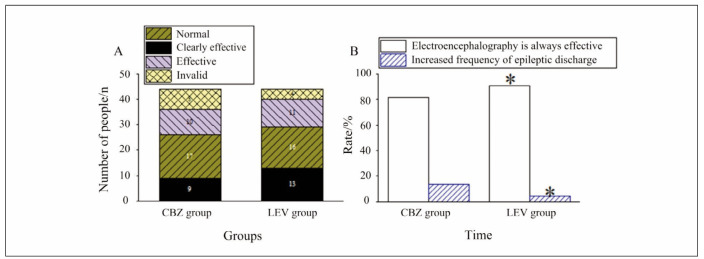
Comparison on TER for EEG of patients after administration. (A: number of patients with different efficacies; B: TER and frequency of epileptiform discharges). Note: * indicated an obvious difference with P<0.05 between the conditions in various groups.

**Figure 7:**
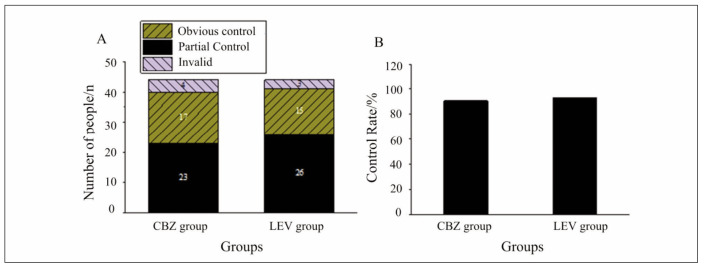
Comparison of frequency of epileptic seizures after administration. (A: number of patients in various control results; B: frequency).

While FIB reductions were noted in both groups, the clinical relevance of this finding remains unclear. It may require further exploration in larger cohorts to determine its implications for bleeding or thrombotic risks in epilepsy patients. Figure 4 Figure 5 Figure 6 Figure 7

CBZ and LEV demonstrated comparable efficacy in controlling seizure frequency, with no significant differences in the proportion of patients achieving significant or partial seizure control. These findings are consistent with previous studies highlighting the effectiveness of CBZ and LEV in managing partialonset epilepsy [9]. However, EEG analysis revealed that LEV had a higher total effective rate (TER) and a lower frequency of epileptiform discharges, suggesting a more favourable neurophysiological impact.

Regarding cognitive function, both drugs significantly improved WAIS-RC scores, which may reflect better seizure control and reduced seizure burden over time. Notably, LEV demonstrated better tolerability concerning anxiety-related side effects, as HAMA scores in the CBZ group remained stable while the LEV group experienced an initial increase followed by normalization. These results suggest that LEV may be a preferable option for patients with pre-existing psychological comorbidities [29] [30].

This study has several limitations. First, the sample size was relatively small, which may limit the generalizability of the findings. Second, the study was conducted over 6 months, and the longer-term effects of CBZ and LEV on coagulation and other parameters remain unclear. Third, while we compared our results with existing literature, the heterogeneity of study designs and methodologies in prior research makes direct comparisons challenging. Finally, the study did not assess the clinical impact of coagulation abnormalities, such as the incidence of bleeding or thrombotic events, which could provide valuable insights into the real-world implications of the observed changes in coagulation parameters.

## Conclusion

This study demonstrates that CBZ has a more pronounced effect on coagulation parameters, specifically PT, APTT, and D-dimer levels, compared to LEV, which shows minimal impact. Both drugs are effective in controlling seizures and improving cognitive function, but LEV may be associated with better psychological tolerability and a safer coagulation profile. These findings emphasize the importance of individualized treatment selection based on patient-specific risks and the need for ongoing monitoring of coagulation function in patients treated with CBZ. Future large-scale, multicenter studies are warranted to confirm these findings and explore their long-term clinical significance.

## Dodatak

### Conflict of interest statement

All the authors declare that they have no conflict of interest in this work.

## References

[1] Azeem G M, Faheem F, Farooq N, Sohail D, et al (2021). Levetiracetam for the Prophylaxis of Migraine in Adults. Cureus J Med Sci.

[2] Chen W Y, Lv X Y, Rong R M, Wu B T, et al (2021). Carbamazepine-induced immune thrombocytopenia confirmed by modified MASPAT test. Transfus Apher Sci.

[3] Cioriceanu I H, Constantin D A, Bobescu E, Marceanu L G, et al (2023). Influence of Clinical Factors on the Quality of Life in Romanian People with Epilepsy-A Follow-Up Study in Real-Life Clinical Practice. J Pers Med.

[4] Felisbino M B, Ziemann M, Khurana I, Okabe J, et al (2021). Valproic acid influences the expression of genes implicated with hyperglycaemia-induced complement and coagulation pathways. Sci Rep.

[5] Gallego M D C, García M A, et al (2022). Acute Carbamazepine intoxication. Neurol Int.

[6] Huang Q J, Liu J G, Shi Z X, Zhu X H, et al (2020). Correlation of MMP-9 and HMGB1 expression with the cognitive function in patients with epilepsy and factors affecting the prognosis. Cell Mol Biol (Noisy-le-grand).

[7] Jawaid W, et al (2023). For the love of all that is holy, stop prescribing sodium valproate and carbamazepine together. Pak J Med Sci.

[8] Kanner A M, Bicchi M M, et al (2022). Antiseizure Medications for Adults With Epilepsy: A Review. JAMA-J Am Med Assoc.

[9] Kharel S, Ojha R, Khanal S, et al (2022). Levetiracetam versus oxcarbazepine as monotherapy in newly diagnosed focal epilepsy: a systematic review and meta-analysis. Brain Behav.

[10] Kośmider K, Kamieniak M, Czuczwar S J, Miziak B, et al (2023). Second Generation of Antiepileptic Drugs and Oxidative Stress. Int J Mol Sci.

[11] Latimer D, Le D, Falgoust E, Ingraffia P, et al (2023). Brivaracetam to Treat Partial Onset Seizures in Adults. Health Psychol Res.

[12] Li X L, Zhou R, Peng H, Peng J, et al (2023). Microglia PKM2 Mediates Neuroinflammation and Neuron Loss in Mice Epilepsy through the Astrocyte C3-Neuron C3R Signaling Pathway. Brain Sci.

[13] Liparoti G, Burchiani B, Mencaroni E, Tripodi D, et al (2022). Individualizing doses of antiepileptic drugs. Expert Opin Drug Metab Toxicol.

[14] Madireddy S, Madireddy S, et al (2023). Therapeutic Strategies to Ameliorate Neuronal Damage in Epilepsy by Regulating Oxidative Stress, Mitochondrial Dysfunction, and Neuroinflammation. Brain Sci.

[15] Myers K A, et al (2022). Genetic Epilepsy Syndromes. Continuum (Minneap Minn).

[16] Novais F, Andrea M, Andrade G, Loureiro S, et al (2022). Intelligence quotient (IQ) as a predictor of epilepsy surgery outcome. Epilepsy Behav.

[17] Oguni H, et al (2022). Epilepsy with myoclonic-atonic seizures, also known as Doose syndrome: Modification of the diagnostic criteria. Eur J Paediatr Neurol.

[18] Olaizola I, Brodde M F, Kehrel B E, Evers S, et al (2023). The Impact of Levetiracetam and Valproate on Platelet Functions-A Double-Blind, Placebo-Controlled Crossover Study. J Clin Med.

[19] Pazarlar B A, Aripaka S S, Petukhov V, Pinborg L, et al (2022). Expression profile of synaptic vesicle glycoprotein 2A, B, and C paralogues in temporal neocortex tissue from patients with temporal lobe epilepsy (TLE). Mol Brain.

[20] Riney K, Bogacz A, Somerville E, Hirsch E, et al (2022). International League Against Epilepsy classification and definition of epilepsy syndromes with onset at a variable age: position statement by the ILAE Task Force on Nosology and Definitions. Epilepsia.

[21] Rugg-Gunn F, Miserocchi A, McEvoy A, et al (2020). Epilepsy surgery. Pract Neurol.

[22] Siniscalchi A, Mintzer S, De Sarro G, Gallelli L, et al (2021). Myotoxicity induced by antiepileptic drugs: could be a rare but serious adverse event?. Psychopharmacol Bull.

[23] Steriade C, Titulaer M J, Vezzani A, Sander J W, et al (2021). The association between systemic autoimmune disorders and epilepsy and its clinical implications. Brain.

[24] Su W L, Lu H T, Li Q D, Tang Z Q, et al (2023). Characteristics of cognition impairment in patients after stroke based on the Wechsler Adult Intelligence Scale-Revised in China. Appl Neuropsychol Adult.

[25] Takeda Y, Sakakibara T, Ogiwara K, Nogami K, et al (2022). Blood coagulation dynamics during adrenocorticotropic hormone therapy in pediatric patients with infantile spasms. Brain Dev.

[26] Thomas S V, Salim S, Jacob N S, Jose M, et al (2022). Language, intelligence, and educational outcomes of adolescents with antenatal exposure to antiseizure medications: Prospective data from the Kerala Registry of epilepsy and pregnancy. Seizure.

[27] Willems L M, van der Goten M, von Podewils F, Knake S, et al (2023). Adverse Event Profiles of Antiseizure Medications and the Impact of Coadministration on Drug Tolerability in Adults with Epilepsy. CNS Drugs.

[28] Xie M G, Qiao J, Wang X F, Zhou J, et al (2022). The cognitive functions and seizure outcomes of patients with low-grade epilepsy-associated neuroepithelial tumors. J Neuro-Oncol.

[29] Yang H J, Shi W X, Fan J J, Wang X S, et al (2023). Transcutaneous Auricular Vagus Nerve Stimulation (ta-VNS) for Treatment of Drug-Resistant Epilepsy: A Randomized, Double-Blind Clinical Trial. Neurotherapeutics.

[30] Ye Z, Guan X M, Shan W Y, Ma B J, et al (2023). Tripchlorolide Inhibits Microglial Activation by Modulating STING-NLRP3 Signaling Pathway in Pilocarpine-Induced Status Epilepticus Model. J Biol Regul Homeost Agents.

